# Applying Machine Learning to Predict Loss to Follow-Up Among People Living With HIV in Haiti Using a National Electronic Medical Record Cohort

**DOI:** 10.3389/ijph.2026.1609496

**Published:** 2026-04-28

**Authors:** Jiaqin Wu, Bryan Shaw, Babatunji Oni, Kurt Jean-Charles, Darwin Dorestan, Marie Bien-Aime, Daphne Compere-Louis, Venise Dorce, Vladimy Jean-Pierre, Marc Elie Joseph, Nancy Rachel Labbe

**Affiliations:** 1 Center for Global Health Practice and Impact (CGHPI), Georgetown University Medical Center, Washington, DC, United States; 2 Solutions S.A., Port-au-Prince, Haiti; 3 Center for Global Health Practice and Impact (CGHPI), Georgetown University Medical Center, Port-au-Prince, Haiti

**Keywords:** electronic medical records, Haiti, HIV, loss to follow-up, machine learning, antiretroviral therapy

## Abstract

**Objectives:**

Loss to follow-up (LTFU) among people living with HIV (PLHIV) remains a major barrier to epidemic control. This study developed machine learning (ML) models to forecast individual risk of LTFU using routine electronic medical record (EMR) data.

**Methods:**

We analyzed data from Haiti’s national EMR database, with 115,822 PLHIV receiving antiretroviral therapy (ART) across 167 health facilities from 2018 to 2024. We trained four ML models, including demographic, clinical, and institutional predictors for LTFU. Model performance was assessed across four quarters using F2-score, recall, precision, ROC-AUC, PR-AUC, and calibration. SHAP values were used to interpret key predictors of LTFU risk.

**Results:**

The CatBoost model trained with weight adjustment performed best across all quarters. The highest F2-score and recall were observed in the first quarter, with modest declines over time. Predictive features with the strongest influence included prior visit and viral load test frequency, ART dispensation patterns, and facility location.

**Conclusion:**

ML models using national EMR data can effectively forecast LTFU risk among PLHIV in Haiti. Incorporating these models into routine care systems can support proactive, tiered interventions to improve retention outcomes.

## Introduction

Retention in care and antiretroviral therapy (ART) is a critical aspect of the global response to the HIV/AIDS epidemic, encompassing the engagement of HIV-positive individuals in ongoing medical care from the point of diagnosis through to the initiation and adherence to ART [1]. As the world adopts a “treat all” strategy, optimizing retention becomes increasingly imperative, particularly for vulnerable groups such as adolescents and young adults who experience poorer outcomes compared to adults across all income settings [2]. In high-income countries, a strategic focus on the cost-effectiveness of retention and re-engagement interventions reflects a nuanced approach to improving HIV outcomes, with interventions more likely to be cost-effective when targeted towards high-risk groups and implemented alongside other high-impact HIV interventions [3].

The situation in low- and middle-income countries (LMIC) presents unique challenges, with substantial loss of patients at every step of the ART care process [4]. Studies indicate that 83% of people living with HIV (PLHIV) have been retained in HIV treatment services after 12 months on ART, but the proportion declined to only 60% after 60 months on treatment [5], increasing healthcare costs [6] as well as the likelihood of virologic failure, transmission, mortality [7], and drug resistance. These effects underscore the urgent need to understand reasons for loss to follow-up (LTFU) and develop effective interventions targeting retention [4]. The disparity in retention strategies between high-income settings and LMIC is significant, with interventions such as decentralization, task-shifting, and differentiated care showing promise in LMIC [8]. However, these regions face socio-structural challenges such as limited healthcare resources, transportation costs, and socio-economic factors that significantly impact patient retention [9].

In Haiti, the landscape of HIV care and retention is defined by its concerted efforts to manage and improve the continuum of HIV care from diagnosis through to ART initiation and retention. The introduction of the iSanté electronic medical record (EMR) system as part of Haiti’s National HIV Quality Management Program represents a significant technological advancement aimed at enhancing the quality of HIV care [10]. Despite these efforts, challenges in retaining patients at every stage of the HIV care cascade persist, with studies revealing that a substantial proportion of individuals diagnosed with HIV are not retained in care or on ART long-term [10]. The variability in retention rates across different regions and among different population groups underscores the complexity of addressing HIV care retention in Haiti [11, 12]. Furthermore, Haiti’s ongoing security and governance crisis and the recent COVID-19 pandemic have created unprecedented challenges to the health system and continuity of chronic disease care [13].

The application of artificial intelligence (AI) in predictive analytics, including the use of machine learning (ML) models in HIV care, has opened new avenues for enhancing patient retention and treatment outcomes in LMIC [14]. Studies utilizing data from EMR registries conducted in Ethiopia [15], Mozambique [16], Nigeria [16–18], South Africa [19, 20], and Tanzania [21] have demonstrated moderately high discrimination from a recent systematic review [22]. However, the authors of this review suggested that future research should prioritize external validation, robust missing data handling, and decision curve analysis, and include sociocultural predictors to improve model robustness [22]. Additionally, others caution that applying models without accounting for socioeconomic variation, often uncaptured during routine EMR data collection, and its impact on predictors, could lead to further marginalization and worse health outcomes for some subpopulations [23]. Despite the promise of AI applications and the increasing use of ML utilizing EMR, there remains a significant evidence gap in their application to ART adherence, particularly in low-resource settings where the burden of disease is greatest.

Our study aims to utilize ML to fill the knowledge gap by developing predictive models tailored to the Haitian context, where socioeconomic factors, healthcare infrastructure, and civil conflict may influence HIV care retention. This study addresses a key knowledge gap in Haiti’s HIV program: while descriptive studies have outlined barriers to retention (e.g., transportation, stigma, and socioeconomic status), no system-wide, data-driven predictive model currently exists to proactively identify clients at high risk for LTFU. Yet the national, routinely collected EMR data contains abundant, longitudinal, and increasingly comprehensive data on over 115,000 PLHIV. By applying ML methods to this robust dataset, our model is intended to support targeted interventions, such as differentiated case management or peer outreach, for clients most likely to disengage. This is especially vital in a resource-limited context like Haiti, where efficient allocation of staff time, medication, and community resources is essential. Moreover, our approach aligns with global best practices advocating for proactive, tailored, and data-informed HIV care, and can serve as a replicable model for other LMIC with similar infrastructure and epidemic profiles.

## Methods

### Program Description

Translating Data and Evidence into Impact (TIDE), implemented by Georgetown’s Center for Global Health Practice and Impact (CGHPI) with Center for Disease Control (CDC) support, has strengthened HIV service delivery across Haiti since 2019 through a comprehensive, client-centered approach to improving retention in care. Operating nationwide, TIDE integrates data-driven community outreach, peer engagement, and decentralized drug dispensing to reduce LTFU and support long-term continuity of ART. Combining human-centered design, biometric tracking, and digital platforms, TIDE has facilitated the return of more than 14,000 clients to treatment and supported the establishment and operation of 56 decentralized drug dispensing points. The program evolved into TIDE Plus (TIDE+), securing multi-year funding to deepen community engagement, enhance differentiated service delivery (DSD), and strengthen national HIV program decision-making. The predictive modeling is designed to complement TIDE’s strategy by identifying individuals at the highest risk of LTFU. This framework will support more proactive and targeted retention interventions, improving program precision and supporting Haiti’s progress toward sustained epidemic control.

### Study Population and Data Sources

Haiti’s national HIV information system is supported by three EMR platforms: iSante, the GHESKIO EMR, and the ZL EMR, implemented across 186 health facilities providing HIV care. All three systems contribute standardized, client-level records to the national HIV data warehouse, SALVH (Surveillance Active Longitudinale du VIH en Haïti), which serves as the central repository for longitudinal HIV surveillance. SALVH harmonizes data inputs from these platforms through a national surveillance protocol initiated at the time of HIV case notification. The system ensures consistent reporting of clinical encounters, ART prescriptions and dispensation, laboratory test results, and core sociodemographic characteristics. SALVH additionally performs deterministic and probabilistic matching to identify duplicate records and generate consolidated, longitudinal treatment histories for individuals receiving care across multiple facilities. The synchronization between the EMR and SALVH is designed to run automatically daily for HIV facilities. In practice, connectivity and power issues mean that, on average, 10% of facilities experience delays of more than 10 days since their last successful update with the central server. SALVH is also used to dispatch data to community-level applications implemented for patient follow-up in different use cases.

For this study, we extracted client-level records for PLHIV from SALVH, representing 167 health facilities across all ten administrative departments. Six standardized SALVH data subsets were used (Supplementary Table S1).

We restricted analyses to EMR data from January 1, 2016 onward to improve longitudinal comparability of care processes and recorded indicators. This period corresponds to an important programmatic transition in Haiti’s HIV response, including national scale-up of routine viral load monitoring in 2015–2016 and adoption of universal ART (“Test and Start”) in July 2016. Because these shifts changed ART eligibility, follow-up, and treatment monitoring practices documented in the EMR, limiting source records to this more standardized care context reduced heterogeneity associated with earlier programmatic periods and improved comparability of derived longitudinal features [24, 25]. Although records from 2016 onward were retained for feature construction, we additionally required evidence of recent engagement between January 1, 2018 and May 1, 2024 to improve analytic quality and reduce the likelihood of including clients with outdated or systematically incomplete records. Specifically, eligible individuals were required to have initiated ART and to have at least two documented ART dispensation events, two clinical encounters, and two VL test results during this period. This restriction served as a verification step to ensure that included clients had sufficient longitudinal information and were plausibly still represented in the active treatment system, rather than reflecting legacy records from individuals no longer engaged in care or affected by data system inconsistencies.

### Data Preparation and Feature Engineering

All predictors were constructed using only information available up to the common index date (Q0: 1 May 2024). Historical EMR records through Q0 were used to generate sociodemographic, facility-level, and longitudinal care-engagement features. No records occurring after 1 May 2024, were used in predictor construction. These baseline predictors were then used to estimate LTFU at four subsequent horizons: Q1 (1 August 2024), Q2 (1 November 2024), Q3 (1 February 2025), and Q4 (1 May 2025). At each horizon, clients were considered LTFU if they had not returned within 28 days of their scheduled medication pickup date. Clients who were documented as deceased, transferred to another clinic, or had voluntarily discontinued ART before the corresponding horizon were excluded from that quarter’s analysis. This design was used to minimize temporal leakage by ensuring that predictors reflected only information that would have been available at the time risk was assessed.

To construct the master dataset, the Patients table was merged with the Institution table using the facility identifier. Age was calculated as of 1 May 2024, and time since ART initiation was derived from the documented enrollment date recorded in the EMRs. The remaining tables contained longitudinal, EMR ID-based time-series records and required transformation prior to analysis.

**FIGURE 1:**
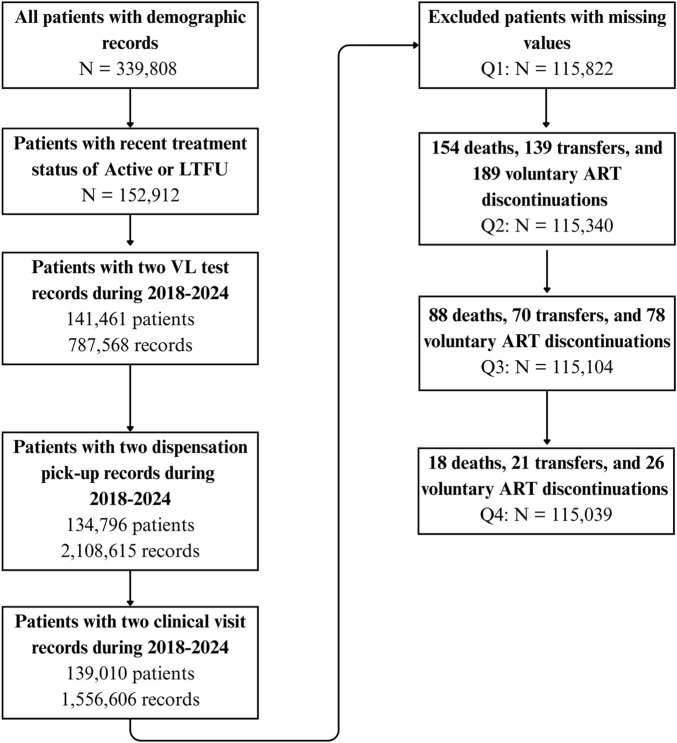
Flow chart for development of cohort with exclusion criteria at each step, Haiti, 2018–2024.

Feature engineering was guided by programmatic relevance, evidence from prior studies, and contextual knowledge from the Haiti HIV program. We generated predictors summarizing prior care engagement, treatment continuity, and monitoring history, including cumulative counts of ART dispensation events, clinical visits, and VL tests. For each service category, timeliness indicators were derived by comparing scheduled and observed service dates to determine whether visits or pickups occurred on time or were delayed. Behavioral indicators related to prior LTFU history were also incorporated to capture longitudinal engagement patterns. LTFU episodes were stratified by duration (1–3, 3–6, 6–12, and >12 months), and the proportion of time spent in each category was calculated for each client. This categorization was chosen to align model outputs with operational follow-up windows used in routine HIV program management, where the primary goal was to identify clients at risk of disengagement within defined near-term intervals rather than estimate exact time to event. Additional plausibility and consistency checks generated binary indicators for clinically incongruent patterns, such as individuals with regular ART pickups but detectable VL results, or those with repeated ART refills but no corresponding VL testing history. Figure 1 presents the development of the analytic cohort at each stage of eligibility assessment and the resulting quarter-specific sample sizes for Q1 through Q4.

After feature generation, the dataset underwent systematic quality assurance to identify data inconsistencies, implausible values, missingness, and outliers. Standardized cleaning procedures were applied across all analytic variables. Implausible entries, such as ART initiation dates preceding an individual’s date of birth, were reviewed and either excluded or recoded according to predefined data-cleaning rules. In alignment with programmatic guidance for active follow-up of clients classified as LTFU, only records with the most recent status change on or after May 1, 2023 were retained, as older status records were considered less likely to reflect current treatment behavior.

Missing-data handling varied by variable type and extent of missingness. For categorical variables with substantial missingness, including marital status and facility type, missing values were not imputed and records were not excluded on that basis. Instead, these observations were retained and coded as a separate category (“Unknown”), allowing these clients to remain in the analytic sample while preserving the distinction between documented and undocumented values. These “Unknown” categories likely reflect routine documentation gaps rather than meaningful social or clinical groupings and were therefore interpreted cautiously. Outside selected categorical variables with substantial undocumented values and a limited number of derived longitudinal features with moderate missingness, most remaining analytic variables had low levels of missing data. Given the very low frequency of missingness in these variables, multiple imputation was not performed, and observations with missing values in any of the remaining analytic fields were removed from the final dataset.

### Model Development

After assembling the analytic dataset, observations were randomly divided into a training set (75%) and a hold-out test set (25%), with stratification by treatment status (LTFU vs. Active) to preserve class proportions. Because LTFU represented the minority class, two strategies were used to address class imbalance during model development. First, class-weighted loss functions were applied to increase the penalty for misclassifying LTFU cases. Second, the Synthetic Minority Oversampling Technique (SMOTE) was applied to the training data to generate synthetic minority-class examples, with the aim of improving representation of clients with LTFU outcomes without simply duplicating existing records.

Given the programmatic need to generate risk estimates to support differentiated service delivery (DSD), we selected ensemble-based tree algorithms that are well-suited to nonlinear relationships, complex interactions, and imbalanced outcome data. Four models were evaluated: Random Forest, XGBoost, LightGBM, and CatBoost. Hyperparameters were optimized using five-fold cross-validation with grid search on the training set. Because several longitudinal indicators were conceptually related and potentially correlated, predictor relationships were examined during data preparation. However, protection against temporal leakage relied primarily on restricting all feature construction to information available before the common index date. Tree-based ensemble methods were considered appropriate in this setting because they are generally robust to correlated predictors in predictive applications. Additional model-development details are provided in Supplementary Material 2, including random seeds, hyperparameter search ranges, final selected hyperparameters, class-weight settings, and the handling of categorical variables across algorithms. For CatBoost, categorical predictors were incorporated using the algorithm’s native handling of categorical features.

Performance evaluation treated LTFU as the positive class. Because the programmatic priority was to identify as many clients at risk of treatment interruption as possible, the F2-score, which weights recall more heavily than precision, was used as the primary model-selection criterion. Recall, precision, ROC-AUC, and PR-AUC were also assessed for each prediction horizon, with ROC-AUC and PR-AUC used as secondary criteria when competing models showed similar F2-score performance. Because programmatic usefulness depends not only on discrimination but also on the agreement between predicted and observed risk, calibration was additionally summarized using appropriate metrics. The best-performing model for each prediction horizon was then evaluated on the held-out test set.

In addition to discrimination metrics, we assessed calibration and operational utility. Calibration was summarized using the Brier score, with lower values indicating better agreement between predicted probabilities and observed outcomes. Because class imbalance can limit the interpretability of ROC-AUC alone, PR-AUC was also reported to better characterize model ranking performance for LTFU. To support interpretation for potential programmatic use, we further evaluated the selected final model using top-risk workload strata (top 1%, 5%, and 10% of clients ranked by predicted risk within each quarter). For each stratum, we summarized the number and proportion of clients flagged, the number of LTFU events captured, recall, positive predictive value (PPV), and the corresponding risk-threshold cutoff.

To support interpretability and programmatic relevance, SHapley Additive exPlanations (SHAP) were used to quantify the marginal contribution of each predictor to model outputs. SHAP values were used to summarize the contribution of individual predictors to CatBoost model outputs and to identify features most strongly associated with LTFU risk, including delayed ART pickups, inconsistent VL testing, and prior LTFU duration. All analyses were conducted in Python (version 3.12). Data management used Pandas and NumPy; model development and evaluation used Scikit-learn, XGBoost, CatBoost, and LightGBM.

### Ethical Statement

The study was reviewed and approved by the Georgetown University Institutional Review Board in the USA (STUDY00009899). In Haiti, ethical review for this study was conducted through Haiti’s Ministère de la Santé Publique et de la Population and the CDC-Haiti (CR00005222). This was a retrospective study of secondary data. All participants were assigned a unique client code in the database, which did not include any personal client identifiers. The study team only had access to de-identified secondary data.

## Results

### Descriptive Analysis

**FIGURE 2:**
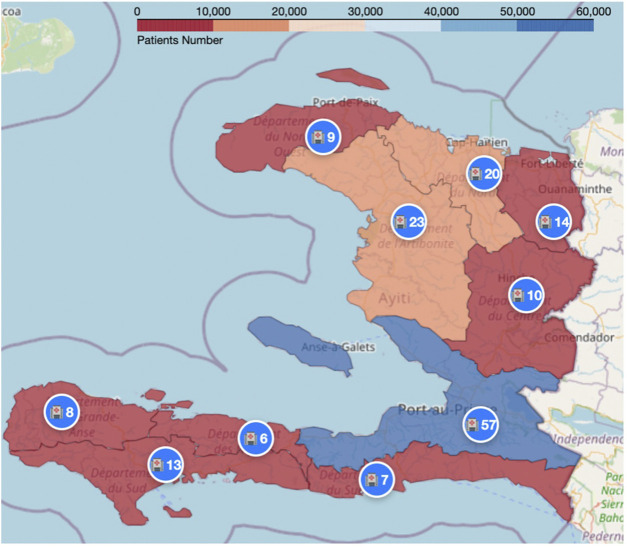
Geographical distribution of clients and facilities, Haiti, 2018–2024.

At the common index date (Q0: May 2024), the final analytic cohort included 115,822 Haitian PLHIV receiving ART across 167 healthcare facilities after cohort eligibility assessment, feature generation, and exclusion of records with incomplete values in the remaining analytic fields. The population at risk was not constant across prediction horizons, as clients who died, transferred to another clinic, or voluntarily discontinued ART were excluded from subsequent quarter-specific analyses. As a result, the proportion of individuals experiencing LTFU was 7.7% in Q1, 8.2% in Q2, 9.7% in Q3, and 12.2% in Q4. Although the analytic baseline cohort was fixed at Q0, quarter-specific risk sets decreased over time as some individuals died, transferred, or voluntarily discontinued ART. Accordingly, Q1 served as the primary reference point for descriptive analyses. Geographically, the Ouest Department, including the capital city of Port-au-Prince, constituted nearly half of the cohort (46.6%) and included the largest number of participating facilities (n = 57), followed by Artibonite (14.0%) and Nord (12.9%) Departments (see Figure 2).

Table 1 provides proportions of the total sample for available sociodemographic and treatment characteristics. A majority of participants were female (62.9%). The age distribution skewed older, with nearly two-thirds of the sample (62.0%) aged ≥40 years and only 4.1% aged <20 years. Over one-third of the sample (37.4%) reported being married or in a partnered relationship, but nearly one-third of the sample’s marital status was missing from the EMR (31.9%). Regarding geographic proximity and contact information, 54.2% of clients resided in the same commune as the facility where they received care. Contact information completeness was high: more than 80% had recorded biometric fingerprint confirmation and a valid residential address, while over 70% provided a functional telephone number. Approximately one-third (31.7%) received ART from health centers with outpatient services only, 13.0% from health centers with inpatient services, 5.2% from dispensaries, and over one-half from other facility types. A small proportion of the sample reported ever having transferred facilities of care (1.2%). Finally, 87.7% of the sample had an undetectable VL on their latest available test.

**TABLE 1:** Sociodemographic characteristics of the study population and by status in quarter 1, Haiti, 2018–2024.

Client & institutional characteristics	Clients (N, %)			P-value
	Total clients (N = 115,822)	Retained in care (N = 106,879)	Lost to follow-up (N = 8,943)	
*Age (years)*	​	​	​	<0.001^a^
Mean age	44.20	44.21	44.07	​
Median age	43.86	43.87	43.42	​
0–19	4,702 (4.1)	4,413 (4.1)	289 (3.2)	​
20–29	10,368 (9.0)	9,486 (8.9)	882 (9.9)	​
30–39	29,000 (25.0)	26,691 (25.0)	2,309 (25.8)	​
40–49	33,921 (29.3)	31,296 (29.3)	2,625 (29.4)	​
≥50	37,831 (32.7)	34,993 (32.7)	2,838 (31.7)	​
*Sex*	​	​	​	<0.001^a^
Female	72,882 (62.9)	67,439 (63.1)	5,443 (60.9)	​
Male	42,940 (37.1)	39,440 (36.9)	3,500 (39.1)	​
*Marital status*	​	​	​	<0.001^a^
Single	20,607 (17.8)	19,256 (18.0)	1,351 (15.1)	​
Married/living together	43,331 (37.4)	41,050 (38.4)	2,281 (25.5)	​
Engaged	5,790 (5.0)	5,499 (5.2)	291 (3.3)	​
Widowed	9,107 (7.9)	8,656 (8.1)	451 (5.0)	​
Unknown	36,987 (31.9)	32,418 (30.3)	4,569 (51.1)	​
*Biometric fingerprint*	​	​	​	0.388
Confirmed record	97,652 (84.3)	90,141 (84.3)	7,511 (84.0)	​
Unconfirmed	18,170 (15.7)	16,738 (15.7)	1,432 (16.0)	​
*Phone number recorded in EMR*	​	​	​	<0.001^a^
Yes	81,403 (70.3)	74,596 (69.8)	6,807 (76.1)	​
No	34,419 (29.7)	32,283 (30.2)	2,136 (23.9)	​
*Address recorded in EMR*	​	​	​	<0.001^a^
Yes	102,807 (88.8)	94,561 (88.5)	8,246 (92.2)	​
No	13,015 (11.2)	12,318 (11.5)	697 (7.8)	​
*Reside in commune where receive antiretroviral therapy*	​	​	​	0.008^a^
Yes	62,793 (54.2)	58,909 (55.1)	3,884 (43.4)	​
No	53,029 (45.8)	47,970 (44.9)	5,059 (56.6)	​
*Facility type where receive antiretroviral therapy*	​	​	​	<0.001^a^
Health center with bed	14,995 (13.0)	14,411 (13.5)	584 (6.5)	​
Health center without bed	36,712 (31.7)	32,142 (30.1)	4,570 (51.1)	​
Dispensary	6,066 (5.2)	5,673 (5.3)	393 (4.4)	​
Other	58,049 (50.1)	54,653 (51.1)	3,396 (38.0)	​
*Ever transferred facilities*	​	​	​	<0.001^a^
Yes	1,397 (1.2)	1,160 (1.1)	237 (2.7)	​
No	114,425 (98.8)	105,719 (98.9)	8,706 (97.4)	​
*Last viral load test result*	​	​	​	<0.001^a^
Detectable	14,261 (12.3)	12,338 (11.5)	1,923 (21.5)	​
Undetectable	101,561 (87.7)	94,541 (88.5)	7,020 (78.5)	​

^a^ Statistically significant difference at p < 0.01 level.

Several statistically significant differences were observed between clients who were LTFU compared to those retained in care (Table 1). Males, clients aged 20–39, and single clients were more likely to be LTFU compared to their counterparts. Clients who were LTFU were more likely to have contact and address details provided in the EMR and to reside in a different commune from the facility where they received ART than those retained in care. Based on the type of facility, those receiving ART from a health center without inpatient services were considerably more likely to be LTFU compared to other facilities. Finally, clients who have reportedly ever transferred facilities and had a detectable VL at the last test were much more likely to be LTFU.

### Model Results and Evaluation

Across all model configurations, the CatBoost model, trained without SMOTE and using class-weighted loss functions, consistently demonstrated the strongest performance based on the F2-score, particularly in Q1 (Table 2). This approach provided the most favorable balance between sensitivity and precision for identifying individuals at elevated risk of becoming LTFU. Models incorporating SMOTE performed uniformly worse than their class-weighted counterparts. In several quarters, SMOTE-based models achieved very high recall but very low precision, indicating that oversampling increased sensitivity to the minority class at the cost of a substantially higher false-positive rate. This pattern suggests that the synthetic minority examples did not adequately capture the heterogeneous longitudinal care trajectories of true LTFU cases, reducing overall discriminative performance relative to class-weighted approaches. Consequently, SMOTE was excluded from the final modeling strategy, and class-weight adjustment was retained as the preferred method for addressing class imbalance.

**TABLE 2:** Comparison of model performance across four prediction quarters, Haiti, 2018–2024.

​	Model performance with weight adjustment						Model performance with SMOTE^a^					
	F2 score	Recall	Precision	ROC-AUC^b^	PR-AUC^c^	Brier^d^	F2 score	Recall	Precision	ROC-AUC^b^	PR-AUC^c^	Brier^d^
*Quarter 1*	​	​	​	​	​	​	​	​	​	​	​	​
Random forest	0.553	0.732	0.279	0.866	0.411	0.126	0.471	0.516	0.349	0.857	0.346	0.081
XGBoost	0.594	0.768	0.311	0.891	0.504	0.108	0.509	0.490	0.601	0.903	0.548	0.049
LightGBM	0.604	0.778	0.319	0.896	0.517	0.105	0.295	1	0.077	0.747	0.200	0.923
CatBoost	0.607	0.773	0.326	0.896	0.516	0.102	0.295	1	0.077	0.528	0.528	0.922
*Quarter 2*	​	​	​	​	​	​	​	​	​	​	​	​
Random forest	0.511	0.687	0.253	0.830	0.345	0.143	0.398	0.435	0.297	0.809	0.288	0.095
XGBoost	0.534	0.716	0.264	0.847	0.389	0.132	0.329	0.309	0.439	0.833	0.358	0.067
LightGBM	0.537	0.713	0.271	0.853	0.400	0.127	0.306	1	0.081	0.617	0.119	0.919
CatBoost	0.543	0.716	0.276	0.850	0.396	0.126	0.306	1	0.081	0.413	0.069	0.919
*Quarter 3*	​	​	​	​	​	​	​	​	​	​	​	​
Random forest	0.537	0.670	0.300	0.827	0.430	0.140	0.502	0.528	0.418	0.804	0.413	0.087
XGBoost	0.557	0.708	0.301	0.840	0.440	0.134	0.507	0.478	0.669	0.861	0.552	0.060
LightGBM	0.580	0.719	0.327	0.853	0.478	0.123	0.348	1	0.097	0.661	0.184	0.903
CatBoost	0.585	0.716	0.339	0.856	0.494	0.119	0.348	1	0.097	0.450	0.087	0.903
*Quarter 4*	​	​	​	​	​	​	​	​	​	​	​	​
Random forest	0.540	0.674	0.302	0.799	0.391	0.168	0.414	0.433	0.350	0.774	0.325	0.123
XGBoost	0.564	0.715	0.306	0.817	0.431	0.163	0.335	0.314	0.459	0.796	0.391	0.095
LightGBM	0.567	0.717	0.309	0.822	0.440	0.159	0.410	1	0.122	0.546	0.143	0.878
CatBoost	0.572	0.735	0.303	0.822	0.435	0.165	0.410	1	0.122	0.546	0.137	0.878

^a^ SMOTE, synthetic minority oversampling technique.

^b^ ROC-AUC, area under the receiver operating characteristic curve.

^c^ PR-AUC, area under the precision-recall curve.

^d^ Brier = a metric to evaluate the accuracy of probabilistic prediction.

In Q1, LightGBM and CatBoost exhibited the highest overall performance among the four tested algorithms. Although LightGBM achieved marginally higher recall (0.778 vs. 0.773), CatBoost demonstrated superior precision (0.326 vs. 0.319), resulting in a slightly higher F2-score (0.607 vs. 0.604). This pattern indicates that CatBoost produced fewer false-positive predictions while maintaining high sensitivity. Based on the F2-score, CatBoost was selected as the optimal model for subsequent evaluation. When evaluated over time, CatBoost maintained the highest F_2_-scores across all quarterly prediction intervals, indicating consistent performance across the prediction horizons evaluated in this study. A gradual decline in performance was observed across quarters, with the F2-score decreasing from 0.607 in Q1 to 0.572 in Q4 and recall declining from 0.773 to 0.735. These reductions likely reflect increased uncertainty associated with predicting outcomes further into the future. Despite this tapering, CatBoost consistently provided the most favorable balance between recall and precision, supporting its potential utility for operational risk stratification within the Haiti TIDE+ program.

PR-AUC values were broadly consistent with the main discrimination metrics and confirmed that the selected CatBoost model maintained useful ranking performance under class imbalance. Across Q1–Q4, CatBoost achieved PR-AUC values of 0.516, 0.396, 0.494, and 0.435, respectively. While LightGBM showed very similar PR-AUC in some quarters, CatBoost remained among the strongest-performing models overall when considered jointly with F2-score, precision, recall, ROC-AUC, and calibration. Table 3 summarizes the workload implications of applying the selected final model at different risk thresholds across the four prediction quarters. PR-AUC and Brier score complemented the primary discrimination metrics by providing additional information on ranking performance under class imbalance and agreement between predicted and observed risk. Brier scores were generally lower in earlier horizons, suggesting better calibration for near-term predictions than for longer-term forecasts. To assess operational utility, we examined top-risk workload strata for the selected final model (Table 3). In Q1, the top 1%, 5%, and 10% of clients ranked by predicted risk captured 10.0%, 39.9%, and 60.1% of LTFU events, respectively, with PPVs of 0.769, 0.616, and 0.464. Comparable tradeoffs were observed across Q2–Q4, indicating that more restrictive thresholds yield higher precision but lower recall, while broader thresholds improve event capture at the cost of greater outreach burden.

**TABLE 3:** Top-risk workload for the selected final model across four prediction quarters, Haiti, 2018–2024.

Quarter	Risk stratum	Client flagged, n (%)	Events captured, n	Recall	PPV	Risk threshold cutoff
Q1	Top 1%	290 (1.0)	223	0.100	0.769	0.955
	Top 5%	1,448 (5.0)	892	0.399	0.616	0.849
	Top 10%	2,896 (10.0)	1,343	0.601	0.464	0.717
Q2	Top 1%	290 (1.0)	202	0.086	0.697	0.922
	Top 5%	1,448 (5.0)	708	0.301	0.489	0.812
	Top 10%	2,896 (10.0)	1,133	0.482	0.391	0.702
Q3	Top 1%	288 (1.0)	225	0.081	0.781	0.928
	Top 5%	1,439 (5.0)	904	0.325	0.628	0.825
	Top 10%	2,878 (10.0)	1,426	0.513	0.495	0.713
Q4	Top 1%	288 (1.0)	213	0.061	0.740	0.915
	Top 5%	1,438 (5.0)	820	0.234	0.570	0.823
	Top 10%	2,876 (10.0)	1,385	0.394	0.482	0.741

In a sensitivity analysis, we assessed whether clients classified as LTFU had subsequent evidence of clinical encounters, ART dispensations, or laboratory records after the date of LTFU labeling. Findings from this assessment supported the validity of the LTFU classification, as individuals labeled as LTFU did not have later records suggesting continued engagement in care.

### Importance of Key Predictor Variables

**FIGURE 3:**
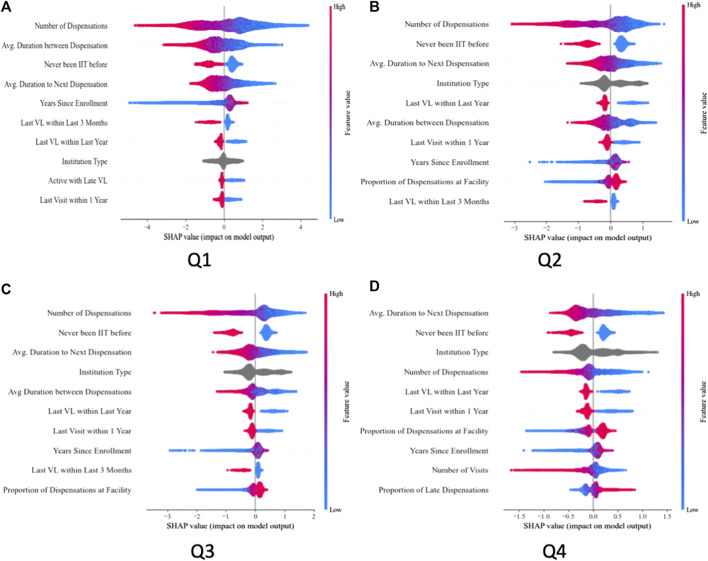
Feature importance of the top 10 predictors from the CatBoost model across four prediction quarters [**(A)**: Q1, **(B)**: Q2, **(C)**: Q3, **(D)**: Q4], Haiti, 2018–2024.

Figure 3 displays the SHAP-derived importance of the top 10 predictors from the CatBoost model across all four prediction quarters. The most influential predictors were behavioral and service utilization indicators, particularly ART dispensation history, clinical visit frequency, and VL testing patterns. Temporal indicators, including time on treatment, recency of care, and service frequency, also contributed substantially to risk stratification. Geographic factors, such as the department of the treatment facility and the client’s commune of residence, further enhanced predictive performance. In contrast, demographic characteristics (e.g., sex) and basic contact indicators (e.g., address or phone number provided in the EMR) showed limited predictive value, as did prior institutional transfers.

The directionality of the associations was consistent with clinical expectations. A greater number of prior ART dispensations was associated with a lower likelihood of subsequent LTFU, and individuals without any history of LTFU were markedly less likely to disengage in the future. Longer intervals between ART initiation and the next scheduled dispensation, likely reflecting clinician confidence in adherence, were also associated with reduced LTFU risk. Recent service utilization emerged as an especially important signal: clinical visits or VL tests within the preceding 3 months or 1 year were protective against disengagement.

Predictor importance varied by prediction horizon. For shorter-term forecasts (e.g., Q1), recent VL testing was among the strongest predictors of retention. For longer-term predictions (e.g., Q4), structural and contextual factors, such as facility characteristics and residential location, gained relative importance, suggesting a gradual shift from proximal behavioral signals to broader contextual determinants as the prediction interval lengthened.

## Discussion

This study demonstrates the feasibility of using ML methods to identify individuals at elevated risk of future LTFU among PLHIV in a low-income setting using routinely collected EMR data. Leveraging over 115,000 records from Haiti’s national EMR system, the CatBoost model trained with class-weight adjustment achieved the strongest overall performance, with the highest F2-score (0.607), recall (0.773), ROC-AUC (0.896), and PR-AUC (0.516) in Q1. This model performed considerably better than similar ML models predicting LTFU in LMIC from a recent review conducted by Kwarah et al. [22], reporting a mean ROC-AUC of 0.668 (SD = 0.066). Although performance declined slightly over longer prediction horizons, this multi-quarter prediction approach offers several advantages. First, it allows for longitudinal monitoring of a client’s risk trajectory over time. Second, forward-looking risk estimates at multiple intervals (e.g., 3-, 6-, 9-, 12-month) provide a practical planning window for follow-up strategies, especially for facilities operating with limited human and financial resources. Together, these features demonstrate how predictive models can move HIV programs beyond reactive tracing efforts toward proactive, data-informed intervention strategies. Predicting future LTFU with high sensitivity is particularly important in Haiti, where health systems are under-resourced and outreach capacity is limited.

This study is among the first national-scale efforts to apply and operationalize an ML-based risk stratification model for HIV retention in a real-world, low-resource context. It contributes to a growing body of research demonstrating the feasibility of applying AI approaches to advance HIV program outcomes in LMIC. Prior studies [15–22] have shown moderate to high performance using similar EMR-based features, but few have successfully integrated prediction models into national health systems or evaluated them across diverse geographies. Our work builds upon these examples by aiming to embed the model directly into Haiti’s national electronic platform (iSanté), enabling weekly generation of LTFU risk scores visible to healthcare providers at selected facilities. This integration represents an important step toward real-time, AI-supported decision-making for public health in fragile settings. Similarly, this aligns with broader calls from WHO and leading scholars to use AI for health systems strengthening in ways that emphasize equity, contextual adaptation, and responsible implementation [26, 27].

The declining performance across longer prediction horizons was expected and supports a practical interpretation of the model. Near-term forecasts were both more accurate and actionable than long-term forecasts, suggesting that the model may be most useful for short-cycle retention planning, while longer-horizon predictions may still help programs prioritize broader monitoring and support strategies. Additional evaluation of top-risk workload strata also highlighted the operational tradeoff between recall and outreach burden: narrower thresholds yielded higher PPV but identified fewer total events, whereas broader thresholds captured more LTFU events at the cost of lower precision. Together with PR-AUC and Brier score results, these findings suggest that the model may support different intervention strategies depending on available staff capacity and follow-up resources.

The top predictors—number and recency of ART dispensations, historical VL test volume, time on treatment—were all indicators already embedded within standard care documentation. These dynamic behavioral and clinical variables outperformed more static demographic factors such as gender, age, or address, which were comparatively weak predictors of LTFU. This pattern is consistent with prior studies [16–19], which found that time-varying EMR features often outperform demographic variables and simpler risk scoring approaches in predicting disengagement from care [19]. Although additional features (e.g., food insecurity, mental health, stigma) may further improve predictive performance, they require non-routine data collection and may be difficult to capture consistently at scale. Recent evidence from Malawi and South Africa has similarly shown that prior disengagement is a robust predictor of future default, reinforcing the need for continuous, tailored follow-up for clients with unstable care patterns [20, 28]. Taken together, these findings suggest that routinely collected EMR data, despite their limitations, contain substantial untapped value for predictive analytics in continuity of care.

Several limitations should be considered when interpreting these findings. Model validation was based on a random train–test split stratified by outcome status, which preserved class balance but provided only internal validation. As a result, transportability across future time periods, facilities, geographic regions, and demographic subgroups remains uncertain. This limitation is particularly relevant because department- and facility-level characteristics were among the more influential predictors, and the analytic sample was unevenly distributed across regions: the Ouest department accounted for nearly half of all client records, whereas departments such as Nippes and Grand’Anse were comparatively underrepresented. These imbalances may reflect differences in data infrastructure, service volume, or care accessibility and may affect model performance across settings. Future work should therefore include temporal validation, facility- or department-level holdout validation, and subgroup-specific performance assessment before broader operational deployment.

In addition, the analysis used quarter-specific binary prediction targets rather than formal time-to-event modeling. Although this design aligns with the programmatic objective of identifying clients at risk within defined operational follow-up windows, it does not fully capture variation in exact event timing or account for right-censoring. Silent transfers and incomplete cross-facility documentation may also have introduced some outcome misclassification by labeling clients as disengaged when they may have continued care elsewhere. Although a post-label record check supported the validity of the LTFU classification within the available data, some visits may still have gone unrecorded because of network disruptions or data-entry backlogs, particularly in rural areas or among more mobile populations. Finally, because EMR histories are generally more complete for clients with sustained engagement in care, the models may perform better for these “data-rich” individuals than for newly initiated clients or others with less complete records. If not carefully monitored, such differences in data completeness could contribute to inequitable model performance and intervention targeting.

This study contributes to the broader and rapidly evolving field of AI in global health. As many LMIC adopt digital health platforms, interest in AI has expanded beyond clinical diagnostics into operational and programmatic domains—including forecasting, resource allocation, and risk stratification. Our findings confirm that even in resource-constrained environments with limited EMR data richness, ML models can offer actionable insights to improve HIV care delivery. Beyond HIV, this work lays the groundwork for broader applications of AI in public health in LMIC. The modeling infrastructure could be adapted for forecasting other programmatic outcomes, such as maternal retention in antenatal care or tuberculosis treatment adherence. Still, the successful deployment of AI in public health requires more than technical performance. Questions of model transparency, data governance, ethical oversight, and integration into routine workflows remain critical—especially in fragile settings like Haiti. Future implementation research should explore how to embed AI models within national systems in a way that supports community health workers, respects client autonomy, and sustains long-term program impact [26].

Overall, this study affirms the feasibility and value of applying ML methods to routine EMR data for predicting future LTFU in a low-income, fragile setting. These findings suggest that ML models using national EMR data may support proactive, risk-informed retention strategies within routine HIV care systems. While challenges remain, including data quality, silent transfers, and equity in risk identification, the model offers a promising tool for early risk stratification. As global interest in AI for health continues to grow, this work contributes a concrete, system-level example of how predictive analytics can support program effectiveness, improve client outcomes, and advance precision public health in LMIC.
