# Supplementary material for: Applying Machine Learning to Predict Loss to Follow-Up Among People Living With HIV in Haiti Using a National Electronic Medical Record Cohort
Source: Int J Public Health. 2026 Apr 28;71:1609496. doi: 10.3389/ijph.2026.1609496 (PMC13160874; doi:10.3389/ijph.2026.1609496)
Supplement: Supplementary file 1 [file Table1.docx]

**Supplementary Material S1.** Data subsets from Surveillance Active Longitudinale du VIH en Haïti/SALVH data warehouse with description of variables used in study, Haiti, 2018-2024

| **Data source** | **Description of variables used in analysis** |
| --- | --- |
| *Patient* | Contains basic personal and clinical information, including date of birth, sex, marital status, institution identification, commune of residence, and antiretroviral therapy enrollment date. |
| *Status* | Contains longitudinal treatment status (e.g., Active, Lost to Follow-up, Transferred, Dead) per client based on electronic medical record identification. |
| *Institution* | Maps institution identification to facility name, geographic location (commune and department), and institution type. |
| *Dispense* | Captures antiretroviral therapy medication dispensing events, including dispense date, next scheduled dispense date, and dispense type. |
| *Visit* | Contains clinical visit records with visit dates, visit types, and scheduled return dates. |
| *Viral load test* | Includes viral load test records with test dates and results (detectable vs. undetectable), linked to electronic medical record identification. |
