# Supplementary material for: Applying Machine Learning to Predict Loss to Follow-Up Among People Living With HIV in Haiti Using a National Electronic Medical Record Cohort
Source: Int J Public Health. 2026 Apr 28;71:1609496. doi: 10.3389/ijph.2026.1609496 (PMC13160874; doi:10.3389/ijph.2026.1609496)
Supplement: Supplementary file 2 [file Table2.docx]

**Supplementary Material S2. Missingness and handling of analytic variables used in Quarter 1 model development, Haiti, 2018–2024 (pre-exclusion analytic sample: n = 134,796)**

| **Variable** | **Variable type** | **Missingness (%)** | **Handling approach** |
| --- | --- | --- | --- |
| Proportion of late viral load tests | Continuous | 11.34 | Observations with missing values were excluded from the final analytic dataset |
| Mean interval between clinical visits | Continuous | 5.01 | Observations with missing values were excluded from the final analytic dataset |
| Proportion of late clinical visits | Continuous | 5.01 | Observations with missing values were excluded from the final analytic dataset |
| Proportion of on-time clinical visits | Continuous | 5.01 | Observations with missing values were excluded from the final analytic dataset |
| Proportion of transfer episodes | Continuous | 1.18 | Observations with missing values were excluded from the final analytic dataset |
| Mean ART dispensation interval | Continuous | 1.18 | Observations with missing values were excluded from the final analytic dataset |
| Proportion of voluntary ART discontinuation episodes | Continuous | 1.18 | Observations with missing values were excluded from the final analytic dataset |
| Proportion of PIT episodes >12 months | Continuous | 1.18 | Observations with missing values were excluded from the final analytic dataset |
| Proportion of PIT episodes 6–12 months | Continuous | 1.18 | Observations with missing values were excluded from the final analytic dataset |
| Proportion of PIT episodes 3–6 months | Continuous | 1.18 | Observations with missing values were excluded from the final analytic dataset |
| Proportion of late ART dispensations | Continuous | 1.18 | Observations with missing values were excluded from the final analytic dataset |
| Proportion of on-time ART dispensations | Continuous | 1.18 | Observations with missing values were excluded from the final analytic dataset |
| Years since enrollment | Continuous | 0.04 | Observations with missing values were excluded from the final analytic dataset |
| Age at enrollment | Continuous | 0.04 | Observations with missing values were excluded from the final analytic dataset |
| Marital status | Categorical | 36.74 | Missing values were coded as “Unknown” and retained in the analysis |
| Facility type | Categorical | 49.36 | Missing values were coded as “Unknown” and retained in the analysis |
