# Supplementary material for: Applying Machine Learning to Predict Loss to Follow-Up Among People Living With HIV in Haiti Using a National Electronic Medical Record Cohort
Source: Int J Public Health. 2026 Apr 28;71:1609496. doi: 10.3389/ijph.2026.1609496 (PMC13160874; doi:10.3389/ijph.2026.1609496)
Supplement: Supplementary file 3 [file Table3.docx]

**Supplementary Material S3. Hyperparameter search space and model-development settings, Haiti, 2018–2024**

| **Model** | **Hyperparameter search space** | **Categorical-variable handling** |
| --- | --- | --- |
| Random Forest | n_estimators: 100, 200, 300; max_depth: 5, 10, 15; min_samples_split: 2, 5; min_samples_leaf: 1, 4 | Trained on preprocessed analytic matrix after categorical encoding |
| XGBoost | n_estimators: 100, 200, 300, 500; max_depth: 4, 6, 8, 10, 12; learning_rate: 0.05, 0.1, 0.01 | Trained on preprocessed analytic matrix after categorical encoding |
| LightGBM | n_estimators: 100, 200, 300, 500; max_depth: 10, -1, 5, 8; learning_rate: 0.05, 0.1, 0.01; num_leaves: 31, 50, 100 | Trained using LightGBM with categorical features specified in the modeling pipeline |
| CatBoost | iterations: 100, 200, 300, 500; depth: 4, 6, 8, 10, 12; learning_rate: 0.05, 0.1, 0.01 | Categorical predictors were incorporated using CatBoost’s native handling of categorical features |

Note: All models were tuned using five-fold cross-validation with grid search on the training set only. To address class imbalance, class-weight adjustment and SMOTE-based oversampling were evaluated in separate modeling pipelines. SMOTE was applied to the training data only after data cleaning and feature generation. Model selection was based primarily on the F2-score, with ROC-AUC and PR-AUC used as secondary criteria when competing models showed similar performance.
