# Supplementary material for: Applying Machine Learning to Predict Loss to Follow-Up Among People Living With HIV in Haiti Using a National Electronic Medical Record Cohort
Source: Int J Public Health. 2026 Apr 28;71:1609496. doi: 10.3389/ijph.2026.1609496 (PMC13160874; doi:10.3389/ijph.2026.1609496)
Supplement: Supplementary file 4 [file Table4.docx]

**Supplementary Material S4. Definitions and coding of variables used in model development, Haiti, 2018–2024**

| **Variable name** | **Variable type** | **Manuscript label** | **Definition / feature engineering description** | **Coding / interpretation** |
| --- | --- | --- | --- | --- |
| Age_At_Enrol | Continuous | Age at enrollment | Age of client at time of ART enrollment | Measured in years |
| Yrs_Since_Enrol | Continuous | Years since enrollment | Time elapsed between ART enrollment date and common index date (Q0) | Measured in years |
| num_disp | Continuous | Number of ART dispensations | Total number of ART dispensation events recorded before Q0 | Count |
| avg_disp | Continuous | Mean ART dispensation interval | Average interval between ART dispensation events before Q0 | Continuous; larger values indicate less frequent dispensing |
| avg_nxt_disp | Continuous | Mean scheduled ART dispensation interval | Average scheduled interval to next ART pickup before Q0 | Continuous |
| num_visit | Continuous | Number of clinical visits | Total number of HIV-related clinical encounters recorded before Q0 | Count |
| avg_nxt_visit | Continuous | Mean scheduled visit interval | Average scheduled interval to next clinical visit before Q0 | Continuous |
| num_hiv | Continuous | Number of HIV laboratory tests | Total number of HIV-related laboratory monitoring records before Q0 | Count |
| same_commune | Binary | Resides in same commune as ART facility | Indicates whether the client’s commune of residence matched the commune of the ART facility | 1 = yes; 0 = no |
| ref_empreinte_Yes | Binary | Biometric identifier recorded | Indicates whether a biometric reference (“empreinte”) was recorded in the EMR | 1 = yes; 0 = no |
| ever_transfert | Binary | Ever transferred care | Indicates whether the client had any recorded transfer between facilities before Q0 | 1 = yes; 0 = no |
| All_actif | Binary | Always active in care | Indicates whether the client remained continuously classified as active in available treatment records before Q0 | 1 = yes; 0 = no |
| Actif_lateVL | Binary | Active with delayed viral load monitoring | Indicates active treatment status with late or overdue viral load monitoring | 1 = yes; 0 = no |
| Actif_detectVL | Binary | Active with detectable viral load | Indicates active treatment status with a recorded detectable viral load result | 1 = yes; 0 = no |
| last_visit_oneyear | Binary | Last clinical visit within past year | Indicates whether the most recent clinical visit occurred within one year before Q0 | 1 = yes; 0 = no |
| last_VL_within_one_year | Binary | Last viral load test within past year | Indicates whether the most recent viral load test occurred within one year before Q0 | 1 = yes; 0 = no |
| last_VL_within_three_months | Binary | Last viral load test within past 3 months | Indicates whether the most recent viral load test occurred within three months before Q0 | 1 = yes; 0 = no |
| last_VL_result_detectable | Binary | Most recent viral load detectable | Indicates whether the latest available viral load result before Q0 was detectable | 1 = yes; 0 = no |
| ValidPhone | Binary | Telephone number recorded in the EMR | Indicates whether a phone contact was recorded in the EMR | 1 = yes; 0 = no |
| ValidAddress | Binary | Address recorded in the EMR | Indicates whether a valid address was recorded in the EMR | 1 = yes; 0 = no |
| Sexe_F | Binary | Sex | Indicates whether client sex is female | 1 = female; 0 = male |
| Marital Status_Engaged | Binary | Married / partnered | Client recorded as married or currently partnered | 1 = yes; 0 = no |
| Marital Status_Previously Engaged | Binary | Previously married / partnered | Client recorded as previously married or partnered (e.g., separated/divorced) | 1 = yes; 0 = no |
| Marital Status_Single | Binary | Single | Client recorded as never married / single | 1 = yes; 0 = no |
| Marital Status_Widowed | Binary | Widowed | Client recorded as widowed | 1 = yes; 0 = no |
| Type of Institution_Dispensary | Binary | Facility type: dispensary | Facility classified as dispensary | 1 = yes; 0 = no |
| Type of Institution_Health Center With Bed | Binary | Facility type: health center with bed | Facility classified as health center with inpatient bed capacity | 1 = yes; 0 = no |
| Type of Institution_Health Center Without Bed | Binary | Facility type: health center without bed | Facility classified as health center without inpatient bed capacity | 1 = yes; 0 = no |
| PIT3months_prop | Derived proportion | Proportion of prior treatment interruption episodes lasting 1–3 months | Proportion of pre-Q0 treatment interruption history falling within 1–3 months | Continuous proportion from 0 to 1 |
| late_disp_prop | Derived proportion | Proportion of late ART dispensations | Proportion of ART dispensation events occurring after the scheduled pickup date | Continuous proportion from 0 to 1 |
| PIT6months_prop | Derived proportion | Proportion of prior treatment interruption episodes lasting 3–6 months | Proportion of pre-Q0 treatment interruption history falling within 3–6 months | Continuous proportion from 0 to 1 |
| PIT1year_prop | Derived proportion | Proportion of prior treatment interruption episodes lasting 6–12 months | Proportion of pre-Q0 treatment interruption history falling within 6–12 months | Continuous proportion from 0 to 1 |
| PIT1moreyears_prop | Derived proportion | Proportion of prior treatment interruption episodes lasting >12 months | Proportion of pre-Q0 treatment interruption history exceeding 12 months | Continuous proportion from 0 to 1 |
| Prop_Disp_Facility | Derived proportion | Proportion of ART dispensations at home facility | Proportion of dispensation events received at the client’s primary / enrolled facility | Continuous proportion from 0 to 1 |
| Prop_Disp_Other | Derived proportion | Proportion of ART dispensations at other facilities | Proportion of dispensation events received outside the client’s primary / enrolled facility | Continuous proportion from 0 to 1 |
| late_visit_prop | Derived proportion | Proportion of late clinical visits | Proportion of clinical visits occurring later than scheduled | Continuous proportion from 0 to 1 |
| prop_late_test | Derived proportion | Proportion of delayed viral load tests | Proportion of viral load tests occurring later than expected based on monitoring schedule | Continuous proportion from 0 to 1 |
| prop_detectable | Derived proportion | Proportion of detectable viral load results | Proportion of viral load results recorded as detectable before Q0 | Continuous proportion from 0 to 1 |

Note: All variables were constructed using information available on or before the common index date (Q0: May 1, 2024). Continuous and derived longitudinal variables were calculated from historical EMR records after data cleaning and feature generation. Binary categorical variables reflect the coding used in the analytic matrix for model development. High-missingness categorical variables were retained using an “Unknown” category where applicable, as described in the supplementary material S2.
